# A three-arm phase III randomised trial assessing, in patients with extensive-disease small-cell lung cancer, accelerated chemotherapy with support of haematological growth factor or oral antibiotics

**DOI:** 10.1054/bjoc.2001.2114

**Published:** 2001-11

**Authors:** J P Sculier, M Paesmans, J Lecomte, O Van Cutsem, J J Lafitte, T Berghmans, G Koumakis, M C Florin, J Thiriaux, J Michel, V Giner, M C Berchier, P Mommen, V Ninane, J Klastersky, the European Lung Cancer Working Party

**Affiliations:** Department of Medicine, Institut Jules Bordet, 1rue Héger-Bordet, Bruxelles, B-1000, Belgium

**Keywords:** small-cell lung cancer, GM-CSF, accelerated chemotherapy

## Abstract

The European Lung Cancer Working Party (ELCWP) designed a 3-arm phase III randomised trial to determine the role of accelerated chemotherapy in extensive-disease (ED) small-cell lung cancer (SCLC). Eligible patients were randomised between the 3 following arms: (A) Standard chemotherapy with 6 courses of EVI (epirubicin 60 mg m^−2^, vindesine 3 mg m^−2^, ifosfamide 5 g m^−2^; all drugs given on day 1 repeated every three weeks. (B) Accelerated chemotherapy with EVI administered every 2 weeks and GM-CSF support. (C) Accelerated chemotherapy with EVI and oral antibiotics (cotrimoxazole). Primary endpoint was survival. 233 eligible patients were randomised. Chemotherapy could be significantly accelerated in arm B with increased absolute dose-intensity. Best response rates, in the population of evaluable patients, were, respectively for arm A, B and C, 59%, 76% and 70%. The response rate was significantly higher in arm B in comparison to arm A (*P* = 0.04). There was, however, no survival difference with respective median duration and 2-year rate of 286 days and 5% for arm A, 264 days and 6% for arm B and 264 days and 6% for arm C. Severe thrombopenia occurred more frequently in arm B but without an increased rate of bleeding. Non-severe infections were more frequent in arm B and severe infections were less frequent in arm C. Our trial failed to demonstrate, in ED-SCLC, a survival benefit of chemotherapy acceleration by using GM-CSF support.   http://www.bjcancer.com


British Journal of Cancer (2001) 85(10), 1444–1451
© 2001 Cancer Research Campaign
doi: 10.1054/ bjoc.2001.2114, available online at http://www.idealibrary.com on IDEAL
http://www.bjcancer.com

### A three-arm phase III randomised trial assessing, in patients with extensive-disease small-cell lung cancer, accelerated chemotherapy with support of haematological growth factor or oral antibiotics

**JP Sculier, M Paesmans, J Lecomte, O Van Cutsem, JJ Lafitte, T Berghmans, G Koumakis, MC Florin, J Thiriaux, J Michel, V Giner, MC Berchier, P Mommen, V Ninane and J Klastersky for the European Lung Cancer Working Party**

Department of Medicine, Institut Jules Bordet, 1rue Héger-Bordet, B-1000 Bruxelles, Belgium

**Summary** The European Lung Cancer Working Party (ELCWP) designed a 3-arm phase III randomised trial to determine the role of accelerated chemotherapy in extensive-disease (ED) small-cell lung cancer (SCLC). Eligible patients were randomised between the 3 following arms: (A) Standard chemotherapy with 6 courses of EVI (epirubicin 60 mg $m^{-2}$, vindesine 3 mg $m^{-2}$, ifosfamide 5 g $m^{-2}$; all drugs given on day 1 repeated every three weeks. (B) Accelerated chemotherapy with EVI administered every 2 weeks and GM-CSF support. (C) Accelerated chemotherapy with EVI and oral antibiotics (cotrimoxazole). Primary endpoint was survival. 233 eligible patients were randomised. Chemotherapy could be significantly accelerated in arm B with increased absolute dose-intensity. Best response rates, in the population of evaluable patients, were, respectively for arm A, B and C, 59%, 76% and 70%. The response rate was significantly higher in arm B in comparison to arm A ($P$ = 0.04). There was, however, no survival difference with respective median duration and 2-year rate of 286 days and 5% for arm A, 264 days and 6% for arm B and 264 days and 6% for arm C. Severe thrombopenia occurred more frequently in arm B but without an increased rate of bleeding. Non-severe infections were more frequent in arm B and severe infections were less frequent in arm C. Our trial failed to demonstrate, in ED-SCLC, a survival benefit of chemotherapy acceleration by using GM-CSF support. © 2001 Cancer Research Campaign http://www.bjcancer.com

**Keywords** : small-cell lung cancer; GM-CSF; accelerated chemotherapy

Despite high response rates and significantly prolonged survival, chemotherapy is associated with only a small percentage of cures in small-cell lung cancer (SCLC) (Paesmans et al, 2000). 5-year overall survival rate rarely exceeds 10% and there are 10 times more long-term survivors in patients with limited disease (LD) than in these with extensive disease. Various attempts have been performed to improve these results, some successful as combination with chest irradiation (Pignon et al, 1992; Warde and Payne, 1992; Luce et al, 1998) but others without significant benefit such as the administration of alternating or sequential combination chemotherapy (Johnson, 1999).

Intensive chemotherapy is an attractive approach to improve survival in SCLC (Sculier and Klastersky, 1989). It can be performed by various types of escalation: increasing the number of active drugs in the combination or the dosage of the administered agents, enhancing the duration of treatment by consolidation and/or maintenance regimens, providing weekly chemotherapy treatments or reducing the intervals between courses of chemotherapy. The European Lung Cancer Working Party (ELCWP) has already assessed some of these concepts in various randomised trials (Sculier et al, 1990, 1993, 1996) and has designed in the early 1990s a trial evaluating accelerated chemotherapy.

Accelerated chemotherapy is an intensive treatment obtained by reducing the intervals between the courses, leading to increased dose-intensity. It has been shown that this approach can be optimised by using haematopoietic growth factors, mainly GM-CSF and G-CSF (Thatcher, 1992). G-CSF (granulocyte-colony stimulating factor) preferentially stimulates neutrophil production and has been shown to reduce the duration of neutropenia following chemotherapy (Lieschke and Burgess, 1992a, b). GM-CSF (granulocyte-macrophage colony stimulating factor) stimulates neutrophil, monocyte and eosinophil production and function. It is associated with more diverse haematological and clinical effects, including improvement of host defence mechanisms.

Both growth factors have been shown able to reduce, in SCLC, the duration of neutropenia induced by chemotherapy and to

*Received 23 April 2001*
*Revised 20 July 2001*
*Accepted 30 July 2001*

*Correspondence to:* JP Sculier

The following institutions participated in the trial: Institut Jules Bordet (JP Sculier, M Paesmans, T Berghmans, P Mommen, J Klastersky,), Brussels, Belgium; Hôpital Saint Pierre (R Sergijsels, V Ninane), Brussels, Belgium; CHU de Charleroi (J Thiriaux, J Lecomte), Charleroi, Belgium; Clinique Saint Luc (O Van Cutsem), Namur, Belgium; CHU de Lille, Hôpital Calmette (JJ Lafitte), Lille, France; Hellenic Cancer Institute (A Efremidis, G Koumakis), Athens, Greece; CH de Douai (MC Florin, E Maetz), Douai, France; CH de Tivoli (J Michel), Tivoli, Belgium; Hospital de Sagunto (V Giner Marco), Valencia, Spain; Hôpital d'Hayange (MC Berchier), Hayange, France; CH de Roubaix (F Kroll), Roubaix, France; CHUA Vésale (D Brohée), Montignies-le-Tilleul, Belgium; CHI de Montfermeil (C Zacharias), Montfermeil, France; IMC Mutualités Socialistes (A Tagnon), Tournai, Belgium; Groupe Médical St Rémi (G Bureau), Reims, France; Hôpital de Warquingnies (P Libert, M Richez), Warquignies, Belgium; CH. de Mons (P Recloux), Mons, Belgium; Clinique de la Louvière (F Fortin), Lille, France; CH Dr Schaffner (J Amourette), Lens, France; Clinique Louis Caty (V Richard), Baudour, Belgium; CH du Pays d'Ath (P Ravez), Ath, Belgium; Klinika radiotherapie a onkologie (J Baumöhl), Kosice, Slovakia; CH de Tourcoing (X Ficheroulle), Tourcoing, France; Cabinet de Pneumologie (Y Watrigant), Tourcoing, France; Hôpital Duchenne (JL Crepin), Boulogne-sur-Mer, France; Hôpital Brugmann (A Drowart), Brussels, Belgium; Hôpital de Braine l'Alleud (C Finet), Braine L'Alleud, Belgium.

reduce the number of febrile neutropenia episodes (Crawford et al, 1991; Trillet-Lenoir et al, 1993, 1995; Hamm et al, 1994). This latter benefit can however be obtained with oral antibiotics prophylaxis such as contrimoxazole as shown in 2 randomised trials (de Jongh et al, 1983; Figueredo et al, 1985).

All these considerations have led the ELCWP to perform in ED SCLC a 3-arm randomised trial, having survival as primary endpoint and comparing to standard 3-weekly chemotherapy, 2-weekly accelerated treatment using the same chemotherapy regimen supported by haematopoietic growth factor (GM-CSF) or by oral prophylactic antibiotics (cotrimoxazole).

### PATIENTS AND METHODS

To be eligible for study entry, patients with pathologically proven SCLC (using World Health Organisation (WHO) classification) had to present with extensive disease. Extensive disease was defined as a disease with distant metastases or as a locoregional disease that could not be locally treated in a single radiotherapy field. The patients should not have received any prior therapy (radiotherapy, chemotherapy or surgery), had to have a Karnofsky performance status (PS) of at least 60, an evaluable or measurable lesion, and no history of prior malignant tumour except non-melanoma skin cancer or in situ carcinoma of the cervix. In addition, they had to have adequate haematologic (WBC count ≥ 4000 $mm^{-3}$ and platelet count ≥ 100 000 $mm^{-3}$), renal (serum creatinine < 1.5 mg $dl^{-1}$) and hepatic (serum bilirubin < 1.5 mg $dl^{-1}$) functions, age less than 75 years, no recent myocardial infection (< 3 months before date of diagnosis), no congestive cardiac failure and cardiac arrhythmia requiring medical treatment, no uncontrolled infectious disease, no history of allergy to cotrimoxazole sulfamides, and no other serious medical or psychologic factors which might prevent adherence to the treatment schedule; patients had to be accessible for follow-up and to provide informed consent. Study protocol was approved by the ethical committee of the hospitals.

Eligible patients were randomised to receive 6 courses of EVI (epirubicin 90 mg $m^{-2}$, vindesine 3 mg $m^{-2}$ and ifosfamide 5 g $m^{-2}$; all drugs given i.v. on day 1) according to 3 different schedules: (A) Standard arm (administration every 3 weeks); (B) Accelerated arm (administration every 2 weeks) with GM-CSF support; (C) Accelerated arm (administration every 2 weeks) with oral antibiotics support (cotrimoxazole). In the 2 accelerated arms, treatment was delayed by one week if on day 15, the neutrophil count was < 1000 $mm^{-3}$ and/or the platelet count < 75 000 $mm^{-3}$. Vindesine and epirubicin were administered by i.v. bolus. Ifosfamide was administered as 24-h i.v. infusion in 1 l NaCl 0.9%. Mesna was infused at a dose ratio 1 g mesna 1 $g^{-1}$ ifosfamide in 11 dextrose 5% NaCl 0.45% over 24 hours, starting at the onset of ifosfamide infusion and followed by an infusion at the ratio 500 mg mesna 1 $g^{-1}$ ifosfamide in 1 1 dextrose 5% NaCl 0.45% over 2 hours. GM-CSF (Leucomax, kindly provided by the Schering-Plough company) was given, as a daily subcutaneous dose of 5 μg $kg^{-1}$, from day 3 through day 13 or until neutrophil count reached ≥ 4000 $mm^{-3}$ after nadir. If neutrophils were < 1000 $mm^{-3}$ on day 13, administration of GM-CSF was continued until reaching this level. Cotrimoxazole (160 mg trimethoprim plus 800 mg sulfamethoxazole) was administered orally every 12 hours from day 3 until the end of the courses of chemotherapy.

Tumour response was assessed after the first 3 courses of chemotherapy and again after completion of therapy. Patients with no change or disease progression after 3 courses were withdrawn from treatment. The total number of courses of chemotherapy was limited to 6 for the responders. In case of recurrence, if the treatment-free interval was greater than 6 months, new courses of EVI were administered. Otherwise, patients went off trial.

The dose-adaptation plan for drugs was as follows: in the standard arm, EVI dosage was reduced by 25% in case of neutrophil nadir < 500 $mm^{-3}$ and/or platelet nadir < 25 000 $mm^{-3}$ and treatment had to be delayed by one week in absence of full haematological recovery (i.e. neutrophil count > 1500 $mm^{-3}$ and platelet count > 100 000 $mm^{-3}$). In the accelerated arms, treatment was delayed by one week if on day 15, neutrophil count was < 1000 $mm^{-3}$ and/or platelet count < 75 000 $mm^{-3}$. In any arm, if the delay between 2 courses of chemotherapy was more than 5 weeks, the patient was taken off treatment. Epirubicin was discontined in cases of cardiac toxicity. WHO criteria were used to report toxicity.

The initial work-up consisted of complete history and physical examination; chest X-ray with computed tomographic (CT) scan and fibreoptic bronchoscopy with biopsy; bone scintigrapy with X-rays of suspected areas; determination of ventricular ejection fraction; liver and adrenal CT scan or echography; brain CT scan; blood chemistries, including complete blood cell count, electrolytes, creatinine, and liver function tests; and ECG. Blood chemistries, ECG, chest X-ray, and clinical examination were repeated before each course. Restaging, including all tests performed during the initial work-up, was repeated after 3 and 6 courses of chemotherapy. After discontinuation of therapy, patients were assessed every 2 months for 6 months and then every 3 months by clinical examination, blood chemistries, and chest X-ray.

Patients were considered as assessable for response if they had completed 3 courses of chemotherapy. Patients' records were evaluated during regular meetings of the group by at least 3 independent observers. Complete response (CR) was defined as the disappearance of all signs of disease, including bronchoscopic, for at least 4 weeks. When some doubtful small lesions remained, the response was called minimal residual disease. In measurable disease, partial response (PR) consisted of a ≥ 50% decrease of the sum of the products of the 2 greatest diameters of all measurable lesions as established by 2 observations no less than 4 weeks apart without the appearance of new lesions or progression of any lesion. Patients with unidimensionally measurable lesions were considered to have assessable disease. In assessable disease, PR was defined as an estimated decrease in tumour size of at least 50%. Progression was defined as an increase of greater than 25% of one or more measurable or assessable lesions or the appearance of new lesions. All other circumstances were classified as no change. Patients with early death due to disease progression before evaluation and those with toxic death due to chemotherapy or early treatment discontinuation due to severe toxicity were considered assessable.

The duration of response was the period between the date of randomisation and the date of first observation of progression or relapsing disease. Survival was measured from randomisation. Survival curves were estimated by the Kaplan–Meier method. The log-rank test was used to compare survival curves, and $P$ values (2-tailed) for testing the null hypothesis for the equality of proportions were calculated using Fisher's exact tests or $\chi^2$ tests. A multivariate analysis for control of prognostic factors was performed by adjusting the data with Cox models for duration of survival and logistic regression models for objective response. $P$ less than 0.05 was considered significant.

The evaluation of chemotherapy intensity was performed by the calculation of 3 dose-related variables. (1) The relative

*© 2001 Cancer Research Campaign* 

dose-intensity (RDI) was defined, for each drug, by the ratio of the received dose divided by the scheduled dose to the actual duration of treatment divided by the scheduled duration. The RDI was expressed in percentage of the projected intensity. (2) The absolute dose-intensity (ADI) was defined as the ratio of the received dose to the actual duration of treatment: it was calculated for each drug and was expressed in milligrams per square metre and time unit. (3) The absolute cumulative dose (CD) did not take time into consideration: it was simply the sum of all received doses. CD was calculated for each drug and was expressed in mg $m^{-2}$. All the formulas were previously reported (Sculier et al, 1993).

Randomisation was stratified by centre, Karnofsky PS (≤ 70 vs ≥ 80), and presence of brain metastases. The procedure was centralised and computerised. Randomisation algorithm used the minimisation technique (Freedman and White, 1976). Treatment assignment was obtained by calling the study data manager. The ELCWP central office for the study coordination and analysis (including the study coordinator, the biostatistician and the data manager) was located at the Jules Bordet Institute in Brussels.

The primary endpoint of the trial was survival. The study was designed to detect a 75% relative increase of median survival time, assumed to be 30 weeks in the control arm (arm A), in one of the experimental arms ($\alpha = 0.05$, $\beta = 0.20$) and required 78 eligible patients in each arm and 195 deaths for statistical analysis (Freedman, 1982). Secondary endpoints were response, toxicity and dose-intensity. An interim analysis for toxicity was performed after registration of the first 30 patients. No interim analysis for survival or response was done.

### RESULTS

243 patients were registered and randomised between April 1993 and April 2000. 10 patients were ineligible (3 in arm A, 3 in arm B and 4 in arm C) for the following reasons: increased serum bilirubin (1), inadequate heart function (2), non-small-cell lung cancer histology (2), existence of a second tumour (2), initial incomplete work-up (1), uncontrolled infection (1) and psychological contra-indication (1). In the 233 eligible patients, 14 were non-assessable for response (2 in arm A, 6 in arm B, an 6 in arm C) for the following reasons: too long delay between 2 courses of chemotherapy (1), early death unrelated to cancer or treatment complications (9), protocol violation (2), death prior to starting treatment (1), no work-up at evaluation (1).

Characteristics of the eligible patients are listed in Table 1. There were 78 patients in arm A (EVI standard), 78 in arm B (accelerated EVI with GM-CSF) and 77 in arm C (accelerated EVI with cotrimoxazole). The 3 arms were well balanced: 84% of the patients were male, 75% had good Karnofsky PS (≥ 80%) and only 8% had locoregionally advanced disease without distant metastases. 20% presented with brain metastases. The median follow-up duration was 4.02 years (range: 0.45–7.49). By time of analysis, 69 in arm A, 74 in arm B and 74 in arm C had died. 14 patients were still alive and one was lost to follow-up.

There was no significant difference in the study arms according to the number of courses administered: 82%, 81% and 79% of the patients received 3 cycles of chemotherapy respectively in arms A, B and C (the figures for 6 courses were 51%, 60% and 52%). GM-CSF and cotrimoxazole could be administered according to treatment schedule in respectively 68% (67% of the courses) and 83% of the patients. The reasons for early discontinuation of GM-CSF were intolerance with asthenia and/or weight loss and/or rash

**Table 1** Patients characteristics

| Characteristics | Arm A EVI standard | Arm B EVI + GM-CSF | Arm C EVI + cotrimoxazole |
|---|---|---|---|
| No of eligible patients | 78 | 78 | 77 |
| Sex | | | |
| Male | 70 | 65 | 61 |
| Female | 8 | 13 | 16 |
| Age (years) | | | |
| Median | 61 | 64 | 61 |
| Range | 37–75 | 35–74 | 37–74 |
| PS | | | |
| 60–70 | 18 | 19 | 20 |
| 80–100 | 60 | 59 | 57 |
| Type of lesions | | | |
| Assessable | 34 | 42 | 42 |
| Measurable | 44 | 36 | 35 |
| Stage | | | |
| III | 4 | 7 | 7 |
| IV | 74 | 71 | 70 |
| Brain metastases | 17 | 14 | 17 |
| Weight loss | | | |
| < 5% | 38 | 41 | 42 |
| ≥ 5% | 29 | 28 | 30 |

(*n* = 17), persistent leucopenia (*n* = 2) and intercurrent complications (*n* = 3). It was never administered in 3 patients. The causes for early discontinuation of cotrimoxazole were lack of compliance (2), allergy (3), digestive intolerance (4), prolonged neutropenia (1) and occurrence of infection (3). There were significantly more treatment delays in the accelerated arms, particularly with arm C: delays occurred during the 3 first courses in 37%, 58% and 87% of the patients respectively for arms A, B and C ($P < 0.001$). For the 6 courses, the figures were respectively 69%, 85% and 97% ($P < 0.001$). The treatment duration was significantly shorter ($P < 0.001$) in patients treated with GM-CSF support: the median duration of the 3 first courses and that of the whole 6 courses were, respectively for arms A, B and C, 65 and 136 days, 45 and 109 days and 63 and 143 days.

The analysis of dose-intensity is summarised in Table 2. There was a non-significant difference between the 3 study arms when the cumulative doses of drugs administered were considered. However, the absolute dose-intensity was significantly higher in arm B and the relative dose-intensity was significantly lower in arm C.

As shown in Table 3, there was no statistically significant difference of response when assessment was performed after the first 3 cycles or if the best response rate was considered. The best rates were obtained in the arm B for the evaluable patients: 69% at 3 courses and 71% as best response. These differences were significant when directly compared to the control arm A which had response rates of respectively 59% ($P = 0.05$) and 59% ($P = 0.04$). There was no significant difference when arms A and C were directly compared ($P = 0.17$ at 3 courses and for best response rate). Overall, we observed only 12 complete responses and 23 minimal residual disease responses while PR was documented in 115 patients. 12 toxic deaths occurred, without difference between the 3 arms. In subgroup analyses (Table 4), we found a significantly higher OR rate in men in favour of arm B and in women in favour of arm C.

 *© 2001 Cancer Research Campaign*

**Table 2** Dose-intensity analysis

| (Median) | Arm A EVI | Arm B EVI + GM-CSF | Arm C EVI + cotrimoxazole | *P* |
|---|---|---|---|---|
| **Cumulative doses (mg m$^{-2}$)** | | Courses 1–3 | | |
| Ifosfamide | 14 700 | 14 700 | 14 600 | 0.48 |
| Vindesine | 8.8 | 8.8 | 8.8 | 0.39 |
| Epirubicin | 264 | 264 | 263 | 0.66 |
| | | Courses 1–6 | | |
| Ifosfamide | 28 900 | 29 400 | 29 200 | 0.14 |
| Vindesine | 17.4 | 17.5 | 17.5 | 0.28 |
| Epirubicin | 524 | 525 | 525 | 0.49 |
| **Absolute dose-intensity (mg/m-2 week$^{-1}$)** | | Courses 1–3 | | |
| Ifosfamide | 1560 | 2250 | 1640 | < 0.001 |
| Vindesine | 0.93 | 1.31 | 0.98 | < 0.001 |
| Epirubicin | 28 | 40 | 29 | < 0.001 |
| | | Courses 1–6 | | |
| Ifosfamide | 1460 | 1840 | 1530 | < 0.001 |
| Vindesine | 0.87 | 1.09 | 0.93 | < 0.001 |
| Epirubicin | 26.1 | 33.6 | 27.5 | < 0.001 |
| **Relative dose-intensity (%)** | | Courses 1–3 | | |
| Ifosfamide | 93 | 90 | 65 | < 0.001 |
| Vindesine | 93 | 87 | 66 | < 0.001 |
| Epirubicin | 93 | 90 | 65 | < 0.001 |
| | | Courses 1–6 | | |
| Ifosfamide | 87 | 73 | 61 | < 0.001 |
| Vindesine | 87 | 73 | 62 | < 0.001 |
| Epirubicin | 87 | 75 | 61 | < 0.001 |

**Table 3** Evaluation of response

| Arm | At 3 courses | | | Best response | | |
|---|---|---|---|---|---|---|
| | A | B | C | A | B | C |
| Assessable patients | 76 | 72 | 71 | 76 | 72 | 71 |
| CR | 2 | 2 | 2 | 4 | 4 | 4 |
| Minimal residual disease | 1 | 1 | 1 | 6 | 9 | 8 |
| PR | 42 | 51 | 47 | 35 | 42 | 38 |
| No change | 5 | 5 | 2 | 5 | 4 | 2 |
| Progression | 16 | 1 | 8 | 16 | 1 | 8 |
| Early death by cancer | 1 | 3 | 2 | 1 | 3 | 2 |
| Toxic death | 5 | 4 | 3 | 5 | 4 | 3 |
| Stop for high toxicity | 4 | 5 | 6 | 4 | 5 | 6 |
| OR rate | 59% | 75% | 70% | 59% | 76% | 70% |
| | | *P* = 0.11 | | | *P* = 0.07 | |
| ITT OR rate | 58% | 69% | 65% | 58% | 71% | 65% |
| | | *P* = 0.32 | | | *P* = 0.25 | |

CR = complete response; PR = partial response; ITT = intent to treat.

**Table 4** Subgroup analyses (intent to treat analysis)

| Arm | Number of patients | | | Best OR rate (%) | | | | Median survival (days) | | | |
|---|---|---|---|---|---|---|---|---|---|---|---|
| | A | B | C | A | B | C | *P* | A | B | C | *P* |
| Men | 70 | 65 | 61 | 59 | 77 | 61 | 0.05 | 265 | 271 | 248 | 0.72 |
| Women | 8 | 13 | 16 | 50 | 39 | 81 | 0.05 | 331 | 204 | 293 | 0.40 |
| PS ≤ 70 | 19 | 18 | 18 | 47 | 61 | 44 | 0.56 | 185 | 212 | 205 | 0.83 |
| PS ≥ 80 | 59 | 60 | 59 | 61 | 73 | 71 | 0.30 | 304 | 279 | 286 | 0.84 |
| Assessable lesions | 34 | 42 | 42 | 59 | 64 | 67 | 0.77 | 265 | 242 | 271 | 0.80 |
| Measurable lesions | 44 | 36 | 35 | 57 | 78 | 63 | 0.14 | 304 | 271 | 219 | 0.99 |
| Weight loss < 5% | 38 | 41 | 42 | 66 | 71 | 64 | 0.81 | 320 | 291 | 236 | 0.32 |
| Weight loss ≥ 5% | 29 | 28 | 30 | 45 | 71 | 67 | 0.09 | 230 | 236 | 248 | 0.77 |
| Age < 60 years | 34 | 30 | 41 | 56 | 77 | 71 | 0.18 | 353 | 279 | 286 | 0.29 |
| Age ≥ 60 years | 44 | 48 | 36 | 59 | 67 | 58 | 0.67 | 230 | 236 | 209 | 0.58 |

© 2001 Cancer Research Campaign

**Table 5** Univariate prognostic factor analysis

| Factors | Best OR rate (%) | *P* | Median survival (day) | *P* |
|---|---|---|---|---|
| Treatment | | | | |
| Arm A (standard) | 58 | | 286 | |
| Arm B (GM-CSF) | 71 | | 264 | |
| Arm C (cotrimoxazole) | 65 | 0.25 | 264 | 0.86 |
| Sex | | | | |
| Male | 65 | | 336 | |
| Female | 60 | 0.58 | 265 | 0.11 |
| PS | | | | |
| ≤ 70 | 51 | | 205 | |
| ≥ 80 | 69 | 0.02 | 286 | < 0.001 |
| Weight loss | | | | |
| < 5% | 67 | | 286 | |
| ≥ 5% | 61 | 0.38 | 242 | 0.31 |
| Type of lesions | | | | |
| Assessable | 64 | | 269 | |
| Measurable | 65 | 0.89 | 267 | 0.90 |
| Age | | | | |
| < 60 years | 68 | | 290 | |
| ≥ 60 years | 62 | 0.41 | 230 | 0.007 |
| Neutrophils | | | | |
| ≤ 7500/mm$^3$ | 66 | | 286 | |
| > 7500/mm$^3$ | 61 | 0.55 | 209 | 0.003 |

OR = objective response.

In the analysis of prognostic factors with respect to response (Table 5), only the Karnofsky performance status was found to be a statistically significant prognostic factor in univariate analysis; treatment arm, sex, weight loss, type of lesions, age and initial neutrophil count were not found to have predictive value.

Duration of response was not statistically different between the arms: the estimated median times (95% confidence interval or CI) were respectively for arm A, B and C, 241 (195–287), 186 (164–206) and 206 (176–236) days ($P = 0.13$).

As shown in Figure 1, there was also no difference in survival between the 3 arms ($P = 0.86$). Overall, the median survival time (95% CI) was 286 (233–349) days for arm A, 264 (220–308) days for arm B and 264 (223–305) days for arm C. 2-year survival rates (95% CI) were respectively 5% (0–11%), 6% (0–12%) and 6% (0–12%). In none of the subgroups analysed (Table 4) there was a significant difference in survival between the 3 arms.

Results of univariate prognostic factors analysis are listed in Table 5. Karnofsky PS, age and initial neutrophil count were associated with a statistically significant prognostic value.

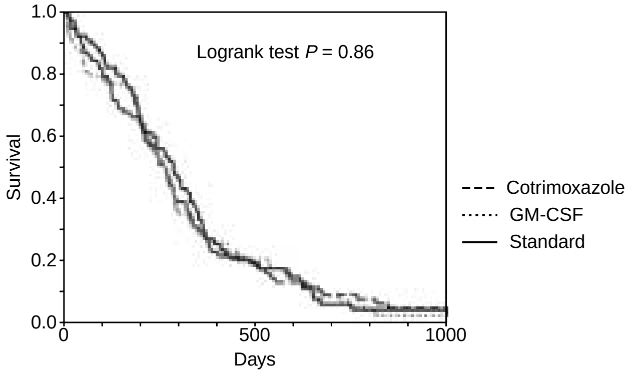

**Figure 1** Survival curves of patients according to regimen ($P = 0.86$)

Multivariate analysis identified as independent prognostic factors age (HR = 1.38, 95% CI 1.03–185; $P = 0.03$), Karnofsky PS (HR = 0.62, 95% CI 0.44–0.88; $P = 0.007$) and neutrophil count (HR = 1.54, 95% CI 1.12–2.10; $P = 0.007$).

Toxicity is summarised in Table 6. The number of patients assessable respectively for haematological and non-haematological toxicities were respectively 74 and 77 in arm A, 74 and 77 in arm B 76 and 72 in arm C. Reasons for non-evaluability were missing evaluations. There was no significant difference between the 3 arms for non-haematological toxicities, including infections and bleedings. Grade III–IV thrombopenia occurred significantly more frequently in accelerated arm B. The difference was not significant for leucopenia nadir but the duration of neutropenia was significantly shorter in arm B during the first course of chemotherapy (median duration of neutropenia: respectively 7, 4 and 7 days for arms A, B and C; $P = < 0.001$). The number of clinical infections observed in the different study arms is listed by grade in Table 7;

**Table 6** Toxicity evaluation (% of patients with at least one grade III or IV during therapy, the most severe episode considered by cycle)

| | Arm | | | |
|---|---|---|---|---|
| Toxicity | A | B | C | *P* |
| Leucopenia | 85 | 84 | 93 | 0.16 |
| Thrombopenia | 16 | 45 | 22 | < 0.001 |
| Nausea and vomiting | 9 | 10 | 14 | 0.63 |
| Diarrhoea | 3 | 4 | 1 | 0.64 |
| Stomatitis | 4 | – | 3 | 0.24 |
| Infections | 18 | 22 | 14 | 0.43 |
| Bleeding | 1 | – | 1 | 0.59 |
| Neurological | 5 | 9 | 7 | 0.64 |
| Respiratory | 4 | 4 | 1 | 0.59 |
| Cardiac | 8 | 4 | 4 | 0.49 |
| Alopecia | 30 | 42 | 42 | 0.23 |
| Renal | 1 | – | – | 0.38 |

*© 2001 Cancer Research Campaign*

**Table 7** Number and grade of infections observed in the different study arms

| | Arm | | |
|---|---|---|---|
| | **A** | **B** | **C** |
| **Number of cycles** | **340** | **360** | **334** |
| Infections | | | |
| Grade I | 10 | 25 | 14 |
| Grade II | 15 | 13 | 17 |
| Grade III | 13 | 21 | 7 |
| Grade IV | 5 | 5 | 3 |
| Total | 43 | 64 | 41 |

the rate of infection increased by 41% in the accelerated arm with GM-CSF, in comparison to the control arm but with fewer grade IV episodes. In the cotrimoxazole arm, the global rate of infections was similar to that of the control arm but with less grade III and IV. 12 toxic deaths were observed: 5 in arm A (3 by complicated febrile neutropenia, 1 by septic shock and 1 by carbonarcosis), 4 in arm B (2 by complicated febrile neutropenia, 1 by septic shock and 1 by haemodynamic pulmonary oedema) and 3 in arm C (2 by complicated febrile neutropenia and 1 by septic shock). Chemotherapy was discontinued for treatment-related reasons in 9 patients: 4 in arm A (cardiac failure, ifosfamide encephalopathy, tumour lysis syndrome and infection) and 5 in arm B (2 by ifosfamide encephalopathy, 2 by infections and 1 by allergic reaction due to GM-CSF).

At relapse, 100 patients received a second-line chemotherapy: 36 in arm A, 33 in arm B and 31 in arm C. The majority of the patients received a combination of a platinum derivative (mainly cisplatin) and etoposide: 31 in arm A, 25 in arm B and 26 in arm C. Only 3 patients (2 in arm A and 1 in arm B) were treated by further cycles of EVI.

### DISCUSSION

Our study showed that the administration of the EVI chemotherapy regimen could be accelerated with GM-CSF support with a significant increase in the relative dose-intensity and with a better response rate. Despite these advantages, the survival was not improved. The same regimen could not be accelerated with oral antibiotics (cotrimoxazole) support.

In the literature, there are only 2 other published trials with a similar design (Steward et al, 1998; Thatcher et al, 2000). In Steward's study (Steward et al, 1998), the V-ICE (vincristine, ifosfamide, carboplatin and etoposide) regimen was randomised to be given over 3 or 4 weeks with or without GM-CSF support. The 3-weekly design was associated with a significantly better survival but the administration of GM-CSF did not result in improved survival, despite higher delivered dose-intensity. Actually, the latter regimen could be safely administered on a 3 weekly basis, without GM-CSF support, and thus it does not represent truly an accelerated chemotherapy. In Thatcher's study (Thatcher et al, 2000), the ACE (adriamycine, etoposide, cyclophosphamide) regimen was administered over a 2-week schedule (with G-CSF support) versus a 3-week one (without growth factor). The experimental arm resulted in significantly increased delivered dose-intensity, complete response rate and survival. Our trial had a design similar to that latter study, with administration of similar individual daily dose size and total dose but we did not achieve a better survival. Our patient population had only extensive disease while in the other studies, there was a majority of patients with limited disease and the number of patients with extensive disease was smaller.

Potential explanations can be offered to understand this difference. Firstly, the patients selection criteria were different. In our trial, we have only included patients with extensive disease. 92% presented with metastatic disease. In the International (Steward et al, 1998) and British (Thatcher et al, 2000) trials, the patients had many good prognostic characteristics and the majority had limited disease. No subgroup analysis has been reported in these 2 papers regarding the patients with extensive disease but the survival distributions as shown on the graphical representations were similar to those of our patients. It is thus possible that the beneficial effect was mainly present in limited disease and that accelerated chemotherapy, allowing better dose-intensity, is effective in that subpopulation of patients with SCLC. This explanation is in accordance with data of studies having shown improved survival with increased absolute dose intensity in limited disease (Arriagada et al, 1993) but not in extensive stage (Pujol et al, 1997).

Our chemotherapy regimen was an association of ifosfamide, epirubicin and vindesine. It was effective to allow acceleration with GM-CSF support but did not contain etoposide and cisplatin, probably the most effective drugs in SCLC as suggested by a meta-analysis that we have recently published (Mascaux et al, 2000). The V-ICE regimen includes etoposide and carboplatin and the ACE contains etoposide. The lack of major differences between the 3 studies in patients with extensive disease makes it unlikely that the drugs chosen might explain the absence of survival improvement. Nevertheless, it should be emphasised that platinum derivatives and etoposide were largely used for salvage chemotherapy in our studies. About one third of the patients were treated at progression with such combinations. Only 17% of the patients received second-line chemotherapy in Thatcher's trial (Thatcher et al, 2000) and no information is given in Steward's paper (Steward et al, 1998) about salvage treatment. It is not impossible that the provided salvage treatment in our study could have improved the survival in the 3 arms.

We have observed an increased response rate with accelerated chemotherapy. When compared to the control arm in assessable patients, the difference was statistically significant. This effect was however not associated with a survival improvement. The response rate that we have obtained was similar to those reported in our prior trials for extensive disease (Paesmans et al, 2000); we had relatively few complete responses: 5.5% CR (meaning complete normalisation of all tests of the work-up) and 10.5% minimal residual disease (meaning the persistence of some characteristics that might be sequellae). This 16% rate is also similar to the rates observed in our prior trials (Paesmans et al, 2000). It is smaller than the rates reported by Steward and Thatcher. The difference between the 3 studies might be explained by the presence of patients with limited disease in the latter trials and by the severity of our criteria to define CR, requiring a full complete work-up including bronchoscopy and CT scan. Indeed, the similarity of survival in extensive disease between the 3 trials suggests that it is more a semantic problem of definition than a true difference. In the subgroup analysis, there was a significant increased response rate for women treated in arm C with cotrimoxazole. We believe that this observation was mainly due to chance (multiple tests were done) and has to be considered very cautiously, the number of female patients being very small.

*© 2001 Cancer Research Campaign* 

We have used GM-CSF as Steward did (Steward et al, 1998); Thatcher (Thatcher et al, 2000) performed his trial with G-CSF. It might be speculated that the choice of GM-CSF rather than G-CSF had contributed to the lack of survival difference for several reasons. It is less well tolerated, leading to early discontinuation as already reported (Hamm et al, 1994). However, 67% of the courses in our GM-CSF arm were performed with the growth factor without problems, allowing a very significant chemotherapy acceleration. In the Steward's trial, where patients were randomised between GM-CSF and placebo in a double-blind design, no significant difference was observed in term of toxicity and treatment discontinuation. An adverse effect of GM-CSF is thus unlikely. In another study (Bunn et al, 1995), where GM-CSF was given concomitantly to chest irradiation, more toxicity in terms of infection and thrombopenia was observed and there was a trend for decreased survival with GM-CSF. An effect of radiotherapy on circulating blood stem cells stimulated by GM-CSF has been advanced to explain the observation. In our trial, radiotherapy was not used; nevertheless, we observed more toxicity in the accelerated arm with GM-CSF. There was a significantly increased haematological toxicity, consisting mainly of more severe thrombopenia, but with fewer bleeding complications. This is a logical consequence of acceleration since the growth factor has no effect on the platelet recovery. A similar toxicity has been observed in all the trials having studied chemotherapy acceleration (Steward et al, 1998; Thatcher et al, 2000).

More infections have also been documented in the GM-CSF arm. In comparison to the control arm, the rate of infection increased by 41%. The difference was restricted to the fewer severe infections and there were no more toxic deaths. This effect is probably due to more overall prolonged myelosuppression, the patients receiving chemotherapy without waiting for full haematological recovery. We could not provide a detailed analysis of the duration of neutropenia because the design of our trial did not include repeated detailed haematological tests during each cycle.

A systematic review of the literature has shown that there are data supporting the administration of consolidation and maintenance chemotherapy (Sculier et al, 1998). In addition, Pujol (Pujol et al, 1997) has shown in a randomised trial that the concentration of chemotherapy over only 4 cycles was associated with shorter survival in comparison to standard 6 cycles treatment, despite the administration of similar theoretical cumulative doses in the 2 arms. This result suggests also that a too-short treatment might not be optimal for SCLC and might explain, partly at least, the lack of effect of our accelerated regimens on survival.

In our study design, we had an arm (arm C) attempting to provide chemotherapy acceleration with support of cotrimoxazole. We made the hypothesis that a prevention of the infectious episodes might allow acceleration of chemotherapy as well as the shortening of neutropenia duration, with GM-CSF. In fact, it was impossible to accelerate chemotherapy without the growth factor support. Nevertheless, we observed fewer severe infections (Table 7) with cotrimoxazole preventive administration, confirming data obtained in trials published in the early 1980s (de Jongh et al, 1983; Figueredo et al, 1985).

In conclusion, the results of our randomised study do not support the practice of chemotherapy acceleration via the support by haematological growth factors in extensive-disease small-cell lung cancer. Despite a better objective response rate, survival was not improved. Our data support the 2000 recommendations of the American Society of Clinical Oncology for the use of haematopoietic CSF in cancer patients (Ozer et al, 2000). Nevertheless, the concept of accelerated chemotherapy might be further tested in patients with better prognosis, as those with limited disease, in appropriately designed randomised controlled trials. A comparison between G-CSF and GM-CSF support might be useful in that type of patients.

### REFERENCES

Arriagada R, Le Chevalier T, Pignon JP, Riviere A, Monnet I, Chomy P, Tuchais C, Tarayre M and Ruffie P (1993) Initial chemotherapeutic doses and survival in patients with limited small-cell lung cancer. *N Engl J Med* **329**: 1848–1852

Bunn PAJ, Crowley J, Kelly K, Hazuka MB, Beasley K, Upchurch C, Livingston R, Weiss GR, Hicks WJ and Gandara DR (1995) Chemoradiotherapy with or without granulocyte-macrophage colony-stimulating factor in the treatment of limited-stage small-cell lung cancer: a prospective phase III randomized study of the Southwest Oncology Group [published erratum appears in *J Clin Oncol* 1995 Nov; **13**(**11**): 2860]: *J Clin Oncol* **13**: 1632–1641

Crawford J, Ozer H, Stoller R, Johnson D, Lyman G, Tabbara I, Kris M, Grous J, Picozzi V and Rausch G (1991) Reduction by granulocyte colony-stimulating factor of fever and neutropenia induced by chemotherapy in patients with small-cell lung cancer. *N Engl J Med* **325**: 164–170

de Jongh CA, Wade JC, Finley RS, Joshi JH, Aisner J, Wiernik PH and Schimpff SC (1983) Trimethoprim/sulfamethoxazole versus placebo: a double-blind comparison of infection prophylaxis in patients with small cell carcinoma of the lung. *J Clin Oncol* **1**: 302–307

Figueredo AT, Hryniuk WM, Strautmanis I, Frank G and Rendell S (1985) Co-trimoxazole prophylaxis during high-dose chemotherapy of small-cell lung cancer. *J Clin Oncol* **3**: 54–64

Freedman LS (1982) Tables of the number of patients required in clinical trials using the logrank test. *Stat Med* **1**: 121–129

Freedman LS and White SJ (1976) On the use of Pocock and Simon's method for balancing treatment numbers over prognostic factors in the controlled clinical trial. *Biometrics* **32**: 691–694

Hamm J, Schiller JH, Cuffie C, Oken M, Fisher RI, Shepherd F and Kaiser G (1994) Doseranging study of recombinant human granulocyte-macrophage colony-stimulating factor in small-cell lung carcinoma. *J Clin Oncol* **12**: 2667–2676

Johnson DH (1999) Management of small cell lung cancer: current state of the art. *Chest* **116**: 525S–530S

Lieschke GJ and Burgess AW (1992a) Granulocyte colony-stimulating factor and granulocyte-macrophage colony-stimulating factor (1). *N Engl J Med* **327**: 28–35

Lieschke GJ and Burgess AW (1992b) Granulocyte colony-stimulating factor and granulocyte-macrophage colony-stimulating factor (2). *N Engl J Med* **327**: 99–106

Luce S, Paesmans M, Berghmans T, Castaigne C, Sotiriou C, Vermylen P and Sculier JP (1998) Revue critique des études randomisées évaluant le rôle de la radiothérapie thoracique adjuvante à la chimiothérapie dans le traitement du cancer bronchique à petites cellules au stade limité. *Rev Mal Respir* **15**: 633–641

Mascaux C, Paesmans M, Berghmans T, Branle F, Lafitte JJ, Lemaitre F, Meert AP, Vermylen P and Sculier JP (2000) A systematic review of the role of etoposide and cisplatin in the chemotherapy of small cell lung cancer with methodology assessment and metaanalysis. *Lung Cancer* **30**: 23–36

Ozer H, Armitage JO, Bennett CL, Crawford J, Demetri GD, Pizzo PA, Schiffer CA, Smith TJ, Somlo G, Wade JC, Wade JL, III, Winn RJ, Wozniak AJ and Somerfield MR (2000) 2000 update of recommendations for the use of hematopoietic colony-stimulating factors: evidence-based, clinical practice guidelines. *J Clin Oncol* **18**: 3558–3585

Paesmans M, Sculier JP, Lecomte J, Thiriaux J, Libert P, Sergysels R, Bureau G, Dabouis G, Van Cutsem O, Mommen P, Ninane V and Klastersky J (2000) Prognostic factors for patients with small cell lung carcinoma: analysis of a series of 763 patients included in 4 consecutive prospective trials with a minimum follow-up of 5 years. *Cancer* **89**: 523–533

Pignon JP, Arriagada R, Ihde DC, Johnson DH, Perry MC, Souhami RL, Brodin O, Joss RA, Kies MS and Lebeau B (1992) A meta-analysis of thoracic radiotherapy for small-cell lung cancer. *N Engl J Med* **327**: 1618–1624

Pujol JL, Douillard JY, Riviere A, Quoix E, Lagrange JL, Berthaud P, Bardonnet-Comte M, Polin V, Gautier V, Milleron B, Chomy F, Chomy P, Spaeth D and Le Chevalier T (1997) Dose-intensity of a four-drug chemotherapy regimen with or without recombinant human granulocyte-macrophage

 *© 2001 Cancer Research Campaign*

colony-stimulating factor in extensive-stage small-cell lung cancer: a multicenter randomized phase III study. *J Clin Oncol* **15**: 2082–2089

Sculier JP and Klastersky J (1989) High-dose chemotherapy of small-cell lung cancer with and without bone marrow transplantation. In *Basic and clinical concepts of lung cancer*, Hansen HH (ed) pp 259–274. Kluwer Academic Publishers: Boston

Sculier JP, Klastersky J, Libert P, Ravez P, Thiriaux J, Lecomte J, Bureau G, Vandermoten G, Dabouis G and Michel J (1990) A randomized study comparing etoposide and vindesine with or without cisplatin as induction therapy for small cell lung cancer. EORTC Lung Cancer Working Party. *Ann Oncol* **1**: 128–133

Sculier JP, Paesmans M, Bureau G, Dabouis G, Libert P, Vandermoten G, Van Cutsem O, Berchier MC, Ries F and Michel J (1993) Multiple-drug weekly chemotherapy versus standard combination regimen in small-cell lung cancer: a phase III randomized study conducted by the European Lung Cancer Working Party. *J Clin Oncol* **11**: 1858–1865

Sculier JP, Paesmans M, Bureau G, Giner V, Lecomte J, Michel J, Berchier MC, Van Cutsem O, Kustner U, Kroll F, Sergysels R, Mommen P and Klastersky J (1996) Randomized trial comparing induction chemotherapy versus induction chemotherapy followed by maintenance chemotherapy in small-cell lung cancer. European Lung Cancer Working Party. *J Clin Oncol* **14**: 2337–2344

Sculier JP, Berghmans T, Castaigne C, Luce S, Sotiriou C, Vermylen P and Paesmans M (1998) Maintenance chemotherapy for small cell lung cancer: a critical review of the literature. *Lung Cancer* **19**: 141–151

Steward WP, von Pawel J, Gatzemeier U, Woll P, Thatcher N, Koschel G, Clancy L, Verweij J, de Wit R, Pfeifer W, Fennelly J, von Eiff M and Frisch J (1998) Effects of granulocyte-macrophage colony-stimulating factor and dose intensification of V-ICE chemotherapy in small-cell lung cancer: a prospective randomized study of 300 patients. *J Clin Oncol* **16**: 642–650

Thatcher N (1992) New perspectives in lung cancer. 4. Haematopoietic growth factors and lung cancer treatment. *Thorax* **47**: 119–126

Thatcher N, Girling DJ, Hopwood P, Sambrook RJ, Qian W and Stephens RJ (2000) Improving survival without reducing quality of life in small-cell lung cancer patients by increasing the dose-intensity of chemotherapy with granulocyte colony-stimulating factor support: results of a British Medical Research Council Multicenter Randomized Trial. Medical Research Council Lung Cancer Working Party. *J Clin Oncol* **18**: 395–404

Trillet-Lenoir V, Green J, Manegold C, von Pawel J, Gatzemeier U, Lebeau B, Depierre A, Johnson P, Decoster G and Tomita D (1993) Recombinant granulocyte colony stimulating factor reduces the infectious complications of cytotoxic chemotherapy. *Eur J Cancer* **29A**: 319–324

Trillet-Lenoir V, Green JA, Manegold C, von Pawel J, Gatzemeier U, Lebeau B, Depierre A, Johnson P, Decoster G and Matcham J (1995) Recombinant granulocyte colony stimulating factor in the treatment of small cell lung cancer: a long-term follow-up. *Eur J Cancer* **31A**: 2115–2116

Warde P and Payne D (1992) Does thoracic irradiation improve survival and local control in limited-stage small-cell carcinoma of the lung? A meta-analysis. *J Clin Oncol* **10**: 890–895

*© 2001 Cancer Research Campaign*
